# Long-term Dose Stability of OnabotulinumtoxinA Injection for Adductor Spasmodic Dysphonia: A 19-Year Single Institution Experience

**DOI:** 10.3389/fsurg.2017.00070

**Published:** 2017-11-29

**Authors:** Paul Paddle, Inna Husain, Christine Moniz, Scott Turner, Ramon Arturo Franco

**Affiliations:** ^1^Department of Otolaryngology, Head and Neck Surgery, Monash Health, Melbourne, VIC, Australia; ^2^Department of Surgery, Faculty Medicine, Nursing and Health Sciences, Monash University, Melbourne, VIC, Australia; ^3^Department of Otorhinolaryngology, Rush University Medical Center, Chicago, IL, United States; ^4^Therapy Resources Management, Somerville, MA, United States; ^5^Department of Otology and Laryngology, Massachusetts Eye and Ear Infirmary, Foxboro, MA, United States; ^6^Department of Otology and Laryngology, Massachusetts Eye and Ear Infirmary, Boston, MA, United States

**Keywords:** laryngeal botox, spasmodic dysphonia, botox dosing, laryngeal dystonia, onabotulinumtoxinA

## Abstract

**Objectives:**

Adductor spasmodic dysphonia (AdSD) is a focal dystonia predominantly involving the laryngeal adductor muscles. AdSD is reported to be a largely non-progressive neurological disorder, though fluctuations in symptom severity do occur. Repeated laryngeal onabotulinumtoxinA (BTX-A) injections are the primary management for AdSD. A number of studies have demonstrated long-term dose stability as evidence of this long-term disease stability.

**Methods:**

A retrospective review was performed on all patients undergoing BTX-A injections for AdSD from April 1994 to September 2013 by a single laryngologist at a tertiary referral laryngology center. Patient demographics, injection doses, use of diazepam and/or lidocaine, and self-reported vocal function were recorded. Multiple linear regression analyses were performed.

**Results:**

83 patients underwent a total of 1,168 injections over 19 years. The mean starting dose was 2.35 MU (0.79 SD). The mean long-term dose was 2.36 MU (0.79 SD). After adjusting for confounders, the change in the relative dose of BTX-A, with every year elapsed since initial dose was 0.13% (95% confidence interval −0.31 to 0.57%), *p* = 0.568.

**Conclusion:**

BTX-A dose is stable over time in our large cohort of patients treated with bilateral thyroarytenoid injections for AdSD.

## Introduction

Spasmodic dysphonia is a chronic, adult-onset neurological disorder that results in involuntary muscle spasms of the vocal folds during task-specific speech (1). Adductor spasmodic dysphonia (AdSD) is the most common subtype occurring in at least 80% of patients with spasmodic dysphonia. It is characterized by spasms of the adductor laryngeal muscles including the thyroarytenoid (TA), interarytenoid, and the lateral cricoarytenoid (LCA) muscles (2). Currently, there is no cure for spasmodic dysphonia.

Temporary management of symptoms is the current standard treatment for AdSD. This can be done by serial laryngeal onabotulinumtoxinA (BTX-A) injections under electromyography guidance in the awake, outpatient setting. First described by Blitzer et al. (2) in the 1980s, this treatment modality is now the primary management strategy recommended by the American Academy of Otolaryngology-Head and Neck Surgery for spasmodic dysphonia (3). The natural progression of symptoms involves a period of breathiness with or without dysphagia starting 24–48 h after the BTX-A injection which is then followed by a period of symptomatic improvement and eventual return of AdSD symptoms (4).

Besides a period of breathiness and possible dysphagia, another disadvantage to this procedure is the temporary nature of the neuromuscular blockade. Due to this, patients can expect to need recurrent laryngeal injections for continued symptomatic relief. In addition, there is a high level of variability between patients regarding the severity of disease, dosage of BTX-A needed to control symptoms, and interval of injections needed (4). Over time, due to variations in disease severity, technique, and the rare occurrence of BTX-A antibodies, dose requirements, and dosing intervals may increase or decrease, or remain largely stable. This leads to difficulties in patient counseling and expectation. Several smaller studies have shown either dose stability over time (5, 6) or a decrease in dose over time (7). More recently, a large 15-year series revealed that, in 39 patients, 41% had a decreasing dose of BTX-A over time, and 59% had increasing doses over the years (8). Essentially, however, there was no significant change in dose over time. This study was designed to quantify any overall long-term BTX-A dosing trend in a large cohort of patients suffering from AdSD.

## Materials and Methods

In our institution, the diagnosis of spasmodic dysphonia is made by reviewing history, perceptual evaluation, and trans-nasal laryngoscopy. Patients are also referred to a movement disorders clinic for an initial neurologist evaluation. Patients also undergo speech laboratory evaluation. A typical response pattern to initial therapeutic BTX-A injection further affirms the diagnosis.

The BTX-A used in this study was Botox^®^ (Allergan Inc., Irvine, CA, USA), at 100 U per vial reconstituted with 4 mL of preservative free sterile 0.9% saline. Percutaneous administration of BTX-A for AdSD has been described in numerous publications. In addition, patients with a history of anxiety or previous coughing or gagging during an injection are offered topical anesthesia in the form of trans-tracheal lidocaine, immediately before the BTX-A injection itself. Occasionally, patients have a preference for systemic anxiolytic instead of the use of trans-tracheal lidocaine and in these cases, 5–10 mg of diazepam is taken orally, at least 45–60 min before the procedure. The majority of patients with adductor type undergo symmetrical, bilateral thyroarytenoid muscle injection at each visit.

The usual starting dose for patients with a diagnosis of AdSD is 2.5 Mouse Units. Patients are instructed to schedule an appointment for repeat injection upon initial return of their symptoms, or sooner if they had no response to the injections. Subsequent doses are adjusted based on patient feedback, balancing therapeutic with adverse effects, and other patient social and professional demands. Key parameters assessed are the period of breathiness (period from onset of breathiness to onset of strong voice in weeks), the period of strong voice (period from offset of breathiness to resumption of functionally significant spasmodic symptoms), and any other significant adverse effect such as dysphagia.

A retrospective chart review was undertaken. All adult patients with a diagnosis of AdSD, undergoing BTX-A injection into the TA–LCA complex, who were treated by the laryngology department at the Massachusetts Eye and Ear Infirmary from April 1994 to September 2013, were included.

Patients were excluded if the diagnosis was not AdSD, if other neurological conditions or movement disorders were present, or if muscle groups other than the TA–LCA complex were injected. Patients injected with preparations of botulinum toxin other than BTX-A were also excluded due to possible dose and potency variability.

Patient demographics, injection doses from commencement of treatment, use of diazepam and/or lidocaine, and vocal function were recorded. Excel 14.4.6 and Stat IC 13.0 were used for data imputation and statistical analysis, respectively.

IRB Approval from the Massachusetts Eye and Ear Infirmary IRB was obtained.

## Results

Eighty-three patients (30.4% males, 69.6% females) underwent a total of 1,168 injections into bilateral TA/LCA muscles for a diagnosis of AdSD, over a period of 19 years. The mean age at first injection was 52.7 years (21–87), and the mean starting dose was 2.35 U. The mean long-term dose was 2.36 U (SD = 0.79). Mean breathiness and good voice duration was 4.26 weeks (SD = 3.74) and 17.0 weeks (SD = 11.9), respectively. On average, patients underwent 14 doses with mean interval between treatments of 26 weeks. 33 (40%) patients received trans-tracheal lidocaine before injection. Nine patients initially received pre-procedural diazepam alone. Of these nine, eight patients (88.88%) switched to lidocaine, leaving one patient (11.1%) who continued to receive pre-procedural diazepam alone (see Table 1).

**Table 1:** Demographic characteristics of study patients and general descriptive statistics.

Characteristic	Results
Age, *mean years (range)*	53.61 (18–87)
Male sex, %	30.4%
Age at first injection, *years (range)*	52.7 (18–87)
Lidocaine use, *n (%)*	33 (40%)
Diazepam use, *n (%)*	1 (11%)
Number switched from diazepam to lidocaine, *n (%)*	8 (89%)
Starting dose, *U*	2.35
Long-term mean dose, *U*	2.36
Mean duration of breathiness, *weeks*	4.26
Mean duration of good voice, *weeks*	17.03
Mean time between injections, *weeks*	26
Mean number of doses per patient, *n*	14

**Figure 1:**
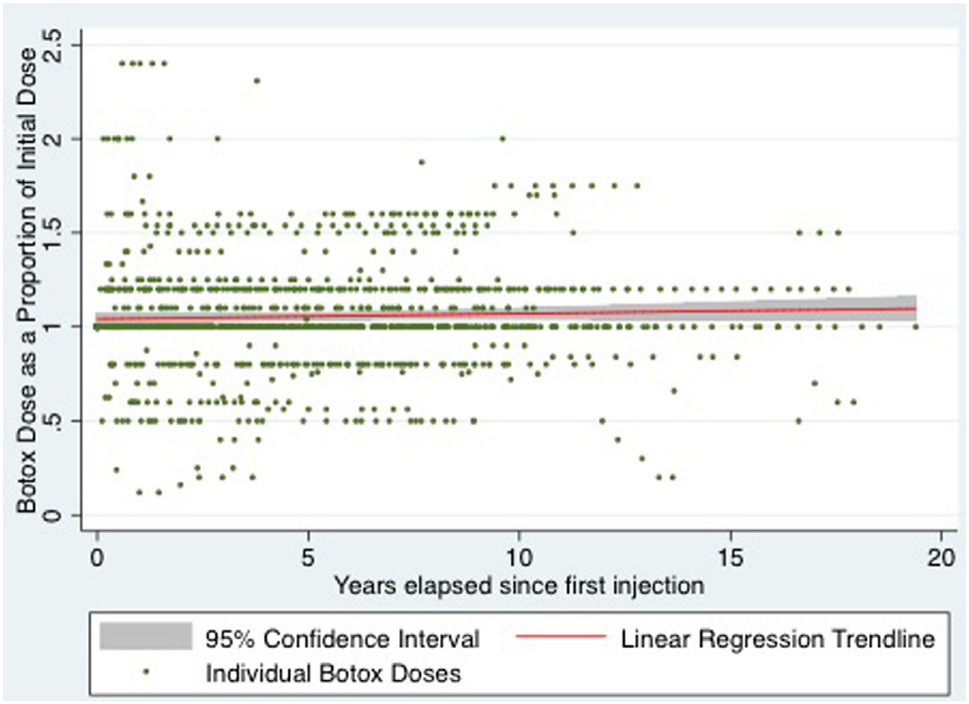
Scatter plot of relative botox dose of all patients over time with linear regression line of best fit and 95% confidence interval.

For statistical analysis, relative dose and time variables were created. Relative dose was obtained by calculating each subsequent dose as a proportion of the initial dose. A relative time variable was calculated as years elapsed since initial injection. Using these relative variables, multiple linear regression was performed, adjusting for the potential confounders of age, sex, diazepam, and lidocaine use. After adjusting for confounders, the change in relative dose of BTX-A, with every year elapsed since initial dose was 0.13% (95% confidence interval −0.31 to 0.57%), *p* = 0.568 (see Figure 1 for all patients).

## Discussion

Spasmodic dysphonia is a chronic neurological disorder of central motor processing that is limited to the laryngeal musculature and characterized by task-specific involuntary muscular spasms (2). In AdSD, the vocal folds are unable to properly coordinate adduction during speech resulting in the voice having a strangled quality. This can lead to a poor quality of life as it can profoundly affect the ability to communicate. Laryngeal BTX-A injections are currently the gold standard treatment for AdSD. They have been demonstrated to show good efficacy in treating AdSD symptoms as well as improving vocal function and thus the quality of life of patients (5).

One useful parameter in the treatment of patients with AdSD is the follow-up of long-term changes in BTX-A dose (6). This parameter can be used to determine possible resistance to the effects of BTX-A as well as chronic irreversible denervation. An increase in BTX-A dose with or without a shortening of treatment intervals and effective weeks can be seen in the former, whereas a decrease in dose with or without prolongation in intervals and effective weeks can be seen in the latter. Our study looking at 19 years, 83 patients, and 1,168 injections demonstrated a very small, statistically non-significant, increase in BTX-A dose over time, with a 95% confidence interval which crossed the null hypothesis. After adjusting for age, sex, diazepam, and lidocaine use, there was found to be a 0.13% increase in dose of BTX-A, per year, since a patient’s initial injection, *p* = 0.568. This equates to an absolute dose of 0.00325 mU. In other words, the observed clinically negligible difference could have occurred by chance, and the true population change in dose per year could be 0%. After adjusting for all confounders, the long-term dose of BTX-A in patients undergoing injection for AdSD is highly stable, and quite likely unchanged.

In addition, we encountered no patients who developed botulinum toxin tolerance or permanent denervation. Notwithstanding subtle intra-patient dose variations, these results confirm the results of previously published studies (5, 6, 8) describing long-term dose stability in patients undergoing serial BTX-A injections for AdSD. This provides helpful information to clinicians to use in counseling patients regarding their long-term care.

To the best of our knowledge, this is the largest single series, both in terms of patient number, and length of follow-up, to analyze BTX-A dosing in AdSD patients. Other strengths of the study include the availability of the doses of BTX-A in all patients since their treatment inception, which provides a more accurate assessment of dose stability over time. The fact that all patients were injected by a single laryngologist using a single technique over these years also helps to strengthen these findings.

## Conclusion

This retrospective review helps to confirm the hypothesis that the dose of BTX-A required in patients receiving injections for AdSD, remains remarkably stable over many years of treatment. While the treatment regimen for individuals is significantly tailored and varied, this over-arching concept of long-term dose stability provides valuable information for both patient and clinician.

## Ethics Statement

Research was conducted after approval from the Institutional Review Board of Massachusetts Eye and Ear Infirmary, Boston, MA, USA.

## Author Contributions

PP: IRB, data collection, data analysis, and manuscript preparation. IH: data collection, analysis, and manuscript preparation. CM and ST: data collection. RF: PI for IRB, subjects recruited from his clinical practice, data analysis, and manuscript preparation review.

## Conflict of Interest Statement

The authors declare that the research was conducted in the absence of any commercial or financial relationships that could be construed as a potential conflict of interest. The reviewer GV and handling editor declared their shared affiliation.
